# International Benchmark for Total Metabolic Tumor Volume Measurement in Baseline ^18^F-FDG PET/CT of Lymphoma Patients: A Milestone Toward Clinical Implementation

**DOI:** 10.2967/jnumed.124.267789

**Published:** 2024-09

**Authors:** Ronald Boellaard, Irène Buvat, Christophe Nioche, Luca Ceriani, Anne-Ségolène Cottereau, Luca Guerra, Rodney J. Hicks, Salim Kanoun, Carsten Kobe, Annika Loft, Heiko Schöder, Annibale Versari, Conrad-Amadeus Voltin, Gerben J.C. Zwezerijnen, Josée M. Zijlstra, N. George Mikhaeel, Andrea Gallamini, Tarec C. El-Galaly, Christine Hanoun, Stephane Chauvie, Romain Ricci, Emanuele Zucca, Michel Meignan, Sally F. Barrington

**Affiliations:** 1 Department of Radiology and Nuclear Medicine, Amsterdam UMC, Cancer Center Amsterdam, Amsterdam, The Netherlands;; 2 LITO, Inserm, Institut Curie, Orsay, France;; 3 Clinic of Nuclear Medicine and PET-CT Centre, Imaging Institute of Southern Switzerland; and EOC, Institute of Oncology Research, Faculty of Biomedical Sciences, Università della Svizzera Italiana, Bellinzona, Switzerland;; 4 Department of Nuclear Medicine, Cochin Hospital, APHP; and Faculté de Médecine, Université Paris Cité, Paris, France;; 5 Nuclear Medicine Unit, Fondazione IRCCS San Gerardo dei Tintori, Monza, Italy;; 6 School of Medicine and Surgery, University of Milano Bicocca, Milan, Italy;; 7 Department of Medicine, St. Vincent’s Hospital Medical School, University of Melbourne, Melbourne, Victoria, Australia;; 8 Centre de Recherche Clinique de Toulouse, Team 9, Toulouse, France;; 9 Department of Nuclear Medicine, Faculty of Medicine and University Hospital Cologne, University of Cologne, Cologne, Germany;; 10 PET & Cyclotron Unit 3982, Copenhagen University Hospital, Copenhagen, Denmark;; 11 Molecular Imaging and Therapy Service, Memorial Sloan Kettering Cancer Center, New York, New York;; 12 Nuclear Medicine Department, Azienda Unità Sanitaria Locale-IRCCS, Reggio Emilia, Italy;; 13 Department of Hematology, Amsterdam UMC, Cancer Center Amsterdam, Amsterdam, The Netherlands;; 14 Department of Clinical Oncology, Guy’s Cancer Centre and School of Cancer and Pharmaceutical Sciences, King’s College London University, London, United Kingdom;; 15 Research and Innovation Department, Antoine Lacassagne Cancer Center, Nice, France;; 16 Department of Hematology, Aalborg University Hospital, Aalborg, Denmark;; 17 Department of Hematology, Odense University Hospital, Odense, Denmark;; 18 Department of Hematology and Stem Cell Transplantation, West German Cancer Center, University Hospital Essen, University of Duisburg-Essen, Essen, Germany;; 19 Medical Physics Division, Santa Croce e Carle Hospital, Cuneo, Italy;; 20 LYSARC, Centre Hospitalier Lyon-Sud, Pierre-Bénite, France;; 21 Oncology Institute of Southern Switzerland; and EOC, Institute of Oncology Research, Faculty of Biomedical Sciences, Università della Svizzera Italiana, Bellinzona, Switzerland; and; 22 King’s College London and Guy’s and St. Thomas’s PET Centre, School of Biomedical Engineering and Imaging Sciences, King’s College London, London, United Kingdom

**Keywords:** total metabolic tumor volume, ^18^F-FDG PET/CT, lymphoma, benchmark

## Abstract

Total metabolic tumor volume (TMTV) is prognostic in lymphoma. However, cutoff values for risk stratification vary markedly, according to the tumor delineation method used. We aimed to create a standardized TMTV benchmark dataset allowing TMTV to be tested and applied as a reproducible biomarker. **Methods:** Sixty baseline ^18^F-FDG PET/CT scans were identified with a range of disease distributions (20 follicular, 20 Hodgkin, and 20 diffuse large B-cell lymphoma). TMTV was measured by 12 nuclear medicine experts, each analyzing 20 cases split across subtypes, with each case processed by 3–4 readers. LIFEx or ACCURATE software was chosen according to reader preference. Analysis was performed stepwise: TMTV1 with automated preselection of lesions using an SUV of at least 4 and a volume of at least 3 cm^3^ with single-click removal of physiologic uptake; TMTV2 with additional removal of reactive bone marrow and spleen with single clicks; TMTV3 with manual editing to remove other physiologic uptake, if required; and TMTV4 with optional addition of lesions using mouse clicks with an SUV of at least 4 (no volume threshold). **Results:** The final TMTV (TMTV4) ranged from 8 to 2,288 cm^3^, showing excellent agreement among all readers in 87% of cases (52/60) with a difference of less than 10% or less than 10 cm^3^. In 70% of the cases, TMTV4 equaled TMTV1, requiring no additional reader interaction. Differences in the TMTV4 were exclusively related to reader interpretation of lesion inclusion or physiologic high-uptake region removal, not to the choice of software. For 5 cases, large TMTV differences (>25%) were due to disagreement about inclusion of diffuse splenic uptake. **Conclusion:** The proposed segmentation method enabled highly reproducible TMTV measurements, with minimal reader interaction in 70% of the patients. The inclusion or exclusion of diffuse splenic uptake requires definition of specific criteria according to lymphoma subtype. The publicly available proposed benchmark allows comparison of study results and could serve as a reference to test improvements using other segmentation approaches.

## Visual Abstract

**Figure:**
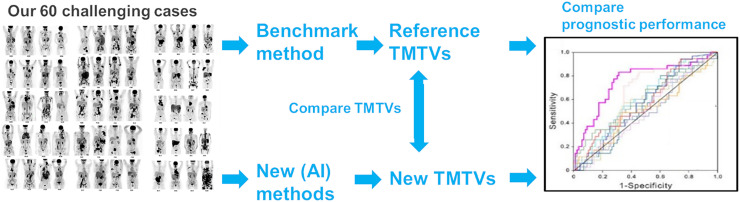

Accurate staging and response assessment are critical for optimal management of lymphoma patients. ^18^F-FDG PET/CT is the current standard for staging and response assessment in ^18^F-FDG–avid lymphomas (1–5).

Pretreatment total metabolic tumor volume (TMTV), measured using ^18^F-FDG PET/CT, provides prognostic information, and TMTV, alone or in combination with other clinical risk factors, outperforms commonly used international prognostic scores (2,6–11). TMTV also offers a more accurate reflection of risk than do traditional staging and CT estimates of bulk (10,12). Prognostic models incorporating TMTV can identify high-risk patients who may require more intensive treatment, and treatment-induced changes in TMTV have been proposed to monitor treatment efficacy (13,14).

However, there are technical challenges to adoption of TMTV (15–18). TMTV assessment requires delineation of metabolically active lymphoma lesions in ^18^F-FDG PET/CT images. The most common segmentation methods use SUV thresholds (e.g., SUV ≥ 2.5 or SUV ≥ 4.0) or a percentage of the SUV_max_ (e.g., 41% SUV_max_) (18). Other methods apply majority-vote approaches, gradient analysis, or artificial intelligence (18,19). Several studies (16,17,20) reported that the prognostic performance of TMTV is similar, irrespective of the methods used, suggesting that the choice of the segmentation approach is not critical. The success of generating visually correct TMTV delineations with common segmentation methods has been explored as well as their discriminative power and manual-editing requirements. The SUV4.0 method was considered the most successful for TMTV delineation in patients with diffuse large B-cell lymphoma and Hodgkin lymphoma (21,22).

Although the prognostic performance of TMTV with different segmentation methods seems comparable, TMTV values vary widely depending on the method. Consequently, a generally applicable TMTV cutoff that discriminates high-risk patients from low-risk patients cannot be used (23). Standardization of TMTV measurements is therefore needed, as stressed in an expert consensus paper from 2019 (18). Successful implementation of TMTV as a quantitative biomarker also depends on availability of a quick, easy, and reproducible measurement method with high accuracy. The method should be robust to variation in PET/CT technology and image reconstruction algorithms to ensure reproducible measurements across imaging sites. The SUV4.0 method appears to be the least affected among several PET image reconstruction protocols (24).

The present study aims to develop and provide an international benchmark dataset to standardize TMTV measurements in lymphoma, thereby allowing TMTV to be used as a reproducible biomarker.

## MATERIALS AND METHODS

### ^18^F-FDG PET/CT Studies

Sixty baseline ^18^F-FDG PET/CT scans from clinical trials were selected (20 follicular, 20 Hodgkin, and 20 diffuse large B-cell lymphoma patients). These scans were acquired as part of the H10 (Hodgkin), AHL2011 (Hodgkin), FOLL12 (follicular), RELEVANCE (follicular), and LNH2007-3B (B-cell) trials (3,25–28). The institutional review board approved these studies, and all subjects provided written informed consent. The scans were selected as representative of the broad range of ^18^F-FDG distributions seen in practice (Supplemental Fig. 1; supplemental materials are available at http://jnm.snmjournals.org) by an international panel of PET/CT lymphoma experts. Scans included less frequently occurring, but not uncommon, uptake patterns, such as focal or diffuse high uptake in the liver and spleen.

### TMTV Assessments

Each panel member assessed 20 cases, balanced between lymphoma subtypes, with each case analyzed by 3–4 readers. TMTVs were measured using LIFEx (29) or ACCURATE (PETRA) software according to reader preference. These software programs were first cross-calibrated by comparing segmentation results for preselection of lesions, with simple removal of physiologic uptake with single clicks and simple addition of tumor uptake by mouse clicks, and by ensuring that TMTVs were equal. One dataset (*n* = 20) was additionally yet independently analyzed using FIJI software (ImageJ) (30).

TMTVs were measured using 4 steps (31). TMTV1 is the automated preselection of lesions using an SUV of at least 4 and a volume of at least 3 cm^3^ with a single-mouse-click removal of physiologic uptake (e.g., brain, bladder). TMTV2 is the additional removal of reactive bone marrow and spleen uptake with single clicks, if required. TMTV3 is the additional manual editing to remove any other physiologic uptake, if required (e.g., in which tumor-related and physiologic uptake was in close proximity, such as the ureter and retroperitoneal nodes included by the software as a single volume). TMTV4, or final TMTV, is the same as TMTV3 except it adds lesions with mouse clicks using an SUV of at least 4 (no volume threshold) as an optional step if the reader considered that this was practical and likely to influence the prognostic assessment.

Reader instructions (Supplemental File 1) indicated which scans to analyze and provided advice about what to include in the TMTV, including focal nodal, splenic, and bone marrow uptake and diffuse uptake in the spleen in the absence of similar reactive changes in the bone marrow (18,32–36). Readers were provided with written manuals or movies illustrating the use of the software tools (Supplemental Files 2–4) and a report form (Supplemental File 5).

### Comparison of TMTVs

For each scan, the reference value for a given TMTV was defined as the median TMTV provided by the 3 or 4 readers. TMTVs were compared with these reference TMTVs for each patient using correlations and difference plots. In addition, we calculated intraclass correlation coefficients between readers for the provided TMTVs. Cases of large discrepancies between the final TMTVs and the reference value, suggesting reader discrepancy, were visually reviewed by an adjudicator, accounting for readers’ comments.

### Data Sharing

The 3D PET/CT images and reference TMTV values corresponding to each step (TMTV1, TMTV2, TMTV3, and TMTV4) of the benchmark are provided and can be downloaded from https://zenodo.org/records/11409717. Coronal and sagittal maximum-intensity projections with overlaid tumor segmentations are provided as a visual indication of lesions included in the final TMTV.

## RESULTS

**FIGURE 1.**
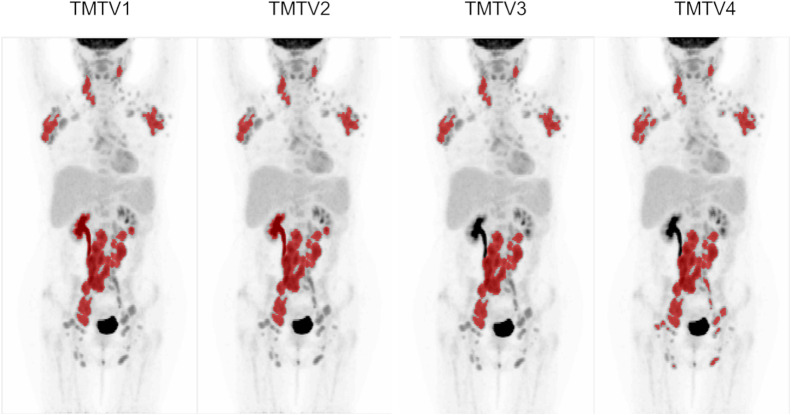
TMTVs for follicular lymphoma patient. TMTV3 shows removal of right renal uptake after manual editing, and TMTV4 included small lesions in pelvis and groin with SUV ≥ 4.0 (volume < 3 cm^3^) added using single mouse clicks.

Figure 1 provides an example of TMTVs obtained at each step. The final TMTV ranged from 8 to 2,288 cm^3^ across scans and readers. In 80% of cases, the final TMTV was identical between readers, and in 87% of cases, it was within 10 cm^3^ or within 10%. TMTV differences were exclusively related to reader interpretation of lesion inclusion or high-uptake region removal for the final TMTV (Table 1). Two readers repeated the analysis with the ACCURATE and LIFEx software tools and reported identical values. Moreover, an additional analysis with FIJI software (30) using the Beth Israel plug-in provided TMTV values within 10% or within 10 cm^3^ of TMTV4 except for the cases listed in Table 1 (Supplemental Fig. 2).

**TABLE 1.** Reported Final TMTVs for 8 Cases with Large Discrepancies in TMTV and Comments by Adjudicator About Causes

	TMTV (cm^3^)				
Patient	Reader 1	Reader 2	Reader 3	Reader 4	Comments from adjudicator
H15	275	129	282	—	Spleen
F02	426	399	456	252	Spleen
F05	1,680	1,706	1,650	567	Spleen
B10	786	788	49	—	Spleen
B16	1,223	546	509	—	Spleen
H11	178	229	137	159	Physiologic uptake removed = 139 cm^3^; manual addition of small multifocal uptake in BM, neck, and retroperitoneal increased to 231 cm^3^
B05	272	321	318	—	Manual editing of myocardial uptake
F09	246	194	196	199	Manual editing of kidneys and ureter

H = Hodgkin lymphoma patient; — = missing data (some cases read by 3 readers only); F = follicular lymphoma patient; B = diffuse large B-cell lymphoma patient; BM = bone marrow.

**FIGURE 2.**
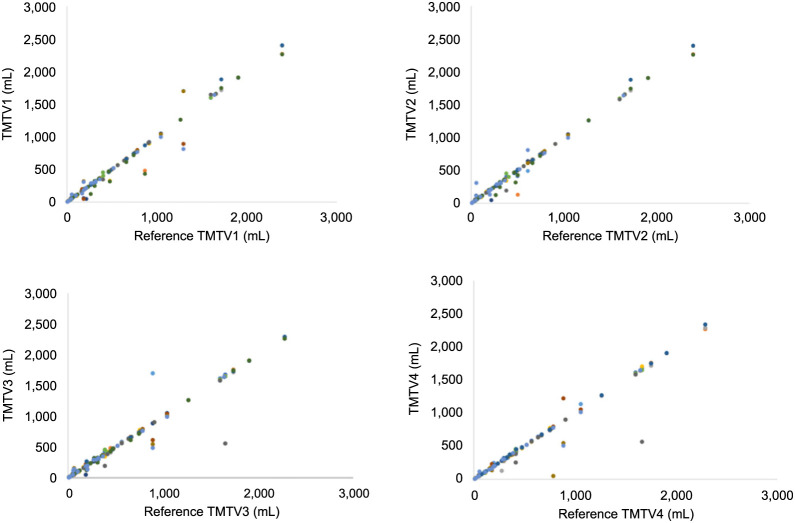
Individual reader TMTV measurements plotted against respective reference values. Different colors for symbols represent different readers.

**FIGURE 3.**
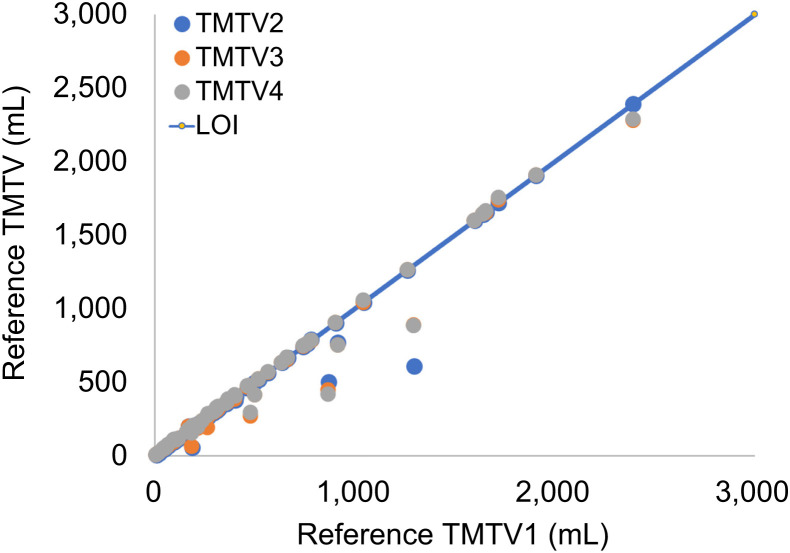
TMTV values for reference or median TMTV2, TMTV3, and TMTV4 against median initial preselection-based TMTV1. LOI = line of identity.

**FIGURE 4.**
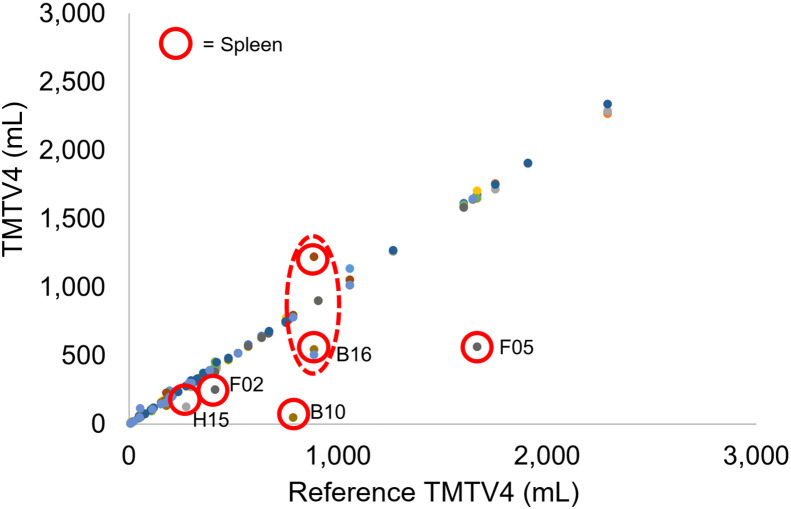
Correlation between TMTV4 assessed by 12 readers (in different colors) and median TMTV4 among readers per scan (reference). Close alignment with line of identity suggests excellent reader agreement. Outliers, indicated by red circles, were all related to interpretation of diffuse splenic uptake. Two outliers enclosed by dashed ellipse are from same scan of B-cell lymphoma patient 16 (B16) with 1 score above and 2 scores below median, in which readers disagreed whether to include spleen or not). For large outliers, patient IDs are indicated. B = diffuse large B-cell lymphoma patient; F = follicular lymphoma patient; H = Hodgkin lymphoma patient.

**FIGURE 5.**
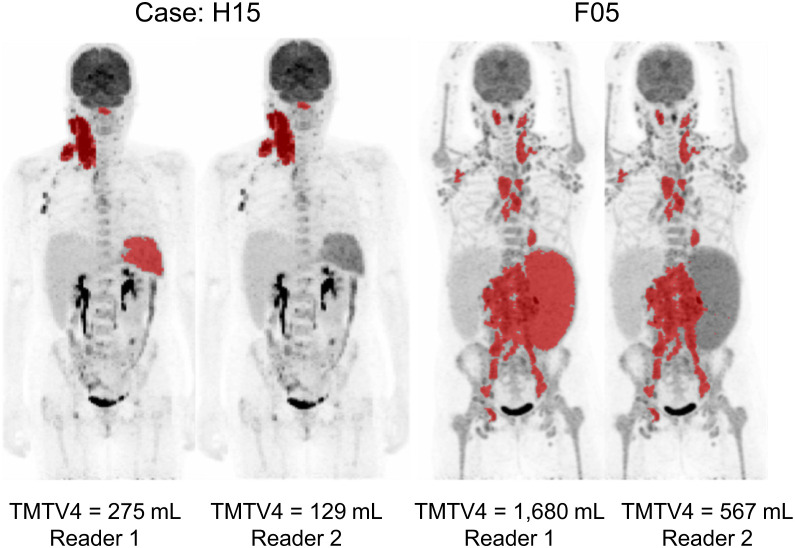
Maximum-intensity projections of Hodgkin lymphoma patient 15 (H15, left 2 panels) and follicular lymphoma patient 5 (F05, right 2 panels) with discrepant TMTV assessment between readers who chose to include or exclude spleen uptake. For other visible lesions, TMTVs were identical.

Figure 2 illustrates the individually reported TMTVs against their reference values (median value among all readers for each patient), for TMTV1–TMTV4, and corresponding difference plots are given in Supplemental Figure 3. Excellent agreement was found between different readers with intraclass correlation coefficients greater than 0.96. There were no significant differences in any of the TMTVs or in any combination of readers (*P* > 0.3 in all cases). Figure 3 illustrates the TMTV obtained for the median TMTV2, TMTV3, and TMTV4 against the median reference TMTV1. In 70% of cases, TMTV4 equaled TMTV1 within 10 cm^3^. Figure 4 shows the final TMTV for individual readers against the final median reference TMTV from 3–4 readers. For the 8 cases with large discrepancies, the final TMTV results are provided in Table 1. For 5 of these 8 cases, large (>25%) differences were exclusively related to decisions about whether to include diffuse splenic uptake (>1.5 times liver uptake) (Fig. 5). The differences between TMTV4 and the other 3 cases ranged from approximately 50 cm^3^ to 100 cm^3^. The agreement between final TMTVs and the median value (per scan) was −10 ± 105 cm^3^ for all scans and 0.8 ± 22.5 cm^3^ after excluding the 5 patients with diffusely increased spleen uptake and 0.4 ± 10.5 cm^3^ after excluding patients listed in Table 1.

## DISCUSSION

Baseline TMTV has emerged as an important prognostic factor in lymphoma subtypes (8,10,18). However, the lack of a standardized TMTV measurement procedure has been an important limitation for use in clinical trials and daily clinical practice. In Hodgkin lymphoma, for example, the reported TMTV cutoffs for optimal prognostication vary between 89 and 268 cm^3^ (23,37).

An international panel of experts was convened to develop an international benchmark for TMTV assessment of baseline ^18^F-FDG PET/CT in lymphoma patients. Considerations for the proposed workflow were that the method is widely available and easy to implement; has a high success rate in providing visually reasonable tumor outlines; is fast and easy to use, that is, with no or minimal manual corrections to generate segmentations; is little sensitive to variations in image quality or reconstruction settings; and demonstrates prognostic performance with a TMTV derived using the proposed method. The segmentation method using an SUV of at least 4.0 was selected as meeting these requirements (21,24,31). To minimize reader variability and to enhance segmentation speed, the workflow starts with automated preselection of lesions using an SUV of at least 4 and a volume of at least 3 cm^3^ and with a single-click removal of normal physiologic uptake. This workflow is available in the software tools in our study and, because of its simplicity, can be easily incorporated in any software.

With the proposed workflow, a final TMTV is obtained including all lesions with an SUV of at least 4 but with removal of high-uptake physiologic regions and manual edits of lesions close to or attached to high-physiologic-tissue regions, such as the brain, bladder, kidneys, and myocardium. These final baseline TMTVs were highly reproducible among multiple readers across 3 lymphoma subtypes, with differences in TMTV of less than 10 cm^3^ or 10% in 85% of the cases. Yet, in about 15% of cases, discrepancies in TMTV were caused by differences in manual editing of healthy tissues with high uptake (e.g., myocardium) or interpretation of diffuse splenic uptake. Hence, manual editing should be done carefully in complicated cases, although its impact on prognostic performance, when TMTV is combined with clinical characteristics, may be small (Supplemental File 6). Most cases with large discrepancies were explained by the interpretation of diffuse splenic uptake. In recent discussions among experts during the 9th International Workshop on PET in Lymphoma and Myeloma in Menton, France, in 2023, it was agreed that focal splenic uptake with an SUV greater than 4 should be included in the TMTV. However, the relevance of diffuse splenic uptake in prognostication was considered uncertain and was recognized to vary by lymphoma subtype, with diffuse reactive uptake in the spleen and bone marrow more commonly seen in Hodgkin lymphoma than in diffuse large B-cell lymphoma or follicular lymphoma (18). Consequently, in future studies, diffuse spleen uptake (and its volume) should be explored as a separate factor for determining prognosis.

### Proposed Use of the Benchmark

The publicly available benchmark, consisting of PET/CT images, previews of the reported segmentations, TMTV4 segmentations, and reference TMTV values, can be used in several ways. First, for technical validation of new and existing clinical or research tools using the SUV4.0 method with minimum volume of 3 cm^3^ to evaluate if the local software can generate TMTVs that are similar (within 10% or 10 cm^3^) to the benchmark values as well as for clinical validation to evaluate if readers can generate comparable TMTVs (within 10% or 10 cm^3^) to international experts. Second, a locally validated benchmark workflow can be applied to new datasets and compared with novel segmentation methods to determine whether these provide improved clinical performance in datasets with available outcome data. Third, the benchmark can help remove the possible confounding effects of segmentation pipelines in multicenter studies or intersite comparisons, provided that each center reports compliance with the benchmark values. Possible examples of how to use the benchmark are given in Supplemental File 7.

### Limitations

The scans were selected to cover the wide range of ^18^F-FDG uptake and distribution seen in lymphoma PET/CT studies and to provide experts with challenging TMTV measurement cases. Consequently, the cases are not necessarily representative of the true prevalence of disease distribution, including more cases with increased diffuse splenic uptake than usually seen in clinical practice. Yet, we felt it was important to include these challenging patterns. The final dataset was approved by an international panel of experts, confirming that the dataset covers the complete range of ^18^F-FDG uptake and distribution experienced in practice. It should be emphasized that the benchmark is not suitable (nor intended) for assessing the clinical performance of a segmentation method. On page 3 of Supplemental File 7, we explain how the benchmark could be used to clinically evaluate new segmentation methods.

Another limitation is that images were collected from existing retrospective clinical trials, and at the time of data collection, Evaluation and Report Language standards were neither fully established nor commonly applied. Because of the age of the datasets, images are likely comparable to the previous Evaluation and Report Language standard 1, although Evaluation and Report Language compliance cannot be stated or proven. However, this does not impact the applicability of the benchmark. The benchmark is intended to allow vendors, software developers, and users to validate their implementation of the benchmark tumor delineation method (SUV4.0) and to show that their tool can generate correct TMTVs by reporting the accuracy and precision of their measurements. The latter technical validation does not rely on the quality of the images used, as the reported benchmark TMTVs are based on these benchmark images. In this way, the benchmark can help to reduce variability in TMTV measurements due to differences in delineation methods or implementations.

This study was designed to provide a benchmark for baseline TMTV measurements, and the degree of uptake in most tumor lesions was higher, typically more than 2-fold, than liver uptake. The proposed segmentation method is unlikely to provide satisfactory TMTVs in interim or end-of-treatment scans, with lower uptake in smaller residual lesions. Other segmentation methods have been suggested for interim PET (38), and assessment of the optimal method for end-of-treatment scans is under evaluation. The SUV4.0 method will not always include all visible tumor regions or tumors. If and to what degree this affects TMTV as a prognostic or predictive factor is largely unexplored and is an intended use of the benchmark. By analyzing a clinical dataset, for example, of patients with classic Hodgkin lymphoma, using the benchmark method as well as any other method that includes low ^18^F-FDG–avid regions or tumors, investigators can start to explore whether including these regions would result in better prognosis or predictions. Such a study was recently performed by Driessen et al. (22), who compared different tumor delineation methods and showed that, in the case of Hodgkin disease, TMTV based on the benchmark method still had the highest clinical prognostic performance among 6 common methods. The proposed benchmark aims at harmonizing TMTV measurements. However, other and better segmentation methods may exist or will be developed, with the expectation that new artificial intelligence–based approaches can provide TMTVs more quickly and reliably. The benchmark method should not be considered a gold standard but rather a universal reference method to test improvements in TMTV measurements or preferably its clinical value as a prognostic marker.

## CONCLUSION

The proposed segmentation method and workflow allowed TMTVs to be generated with high reproducibility among readers and software tools, with minimal reader interaction in 70% of cases. The inclusion or exclusion of diffuse splenic uptake requires further study to define specific criteria that might vary according to lymphoma subtype. The TMTV dataset is publicly available as a benchmark to allow imaging departments, software developers, and vendors to implement and validate the workflow. The proposed TMTV measurement workflow allows comparison, sharing, and pooling of study results. Moreover, it could serve as a reference to test potential improvements in the measurement of TMTV using other or artificial intelligence approaches. On the basis of our findings, we recommend that the SUV4.0 method should be included or at least tested in future clinical trials.

## DISCLOSURE

Sally Barrington acknowledges support from the National Institute for Health and Care Research (NIHR) (RP-2016-07-001). This work was also supported by core funding from the Wellcome/EPSRC Centre for Medical Engineering at King’s College London (WT203148/Z/16/Z). The views expressed are those of the authors and not necessarily those of the NHS, the NIHR, or the Department of Health and Social Care. Heiko Schöder was supported in part by NIH/NCI Cancer Center Support Grant P30 CA008748. Ronald Boellaard is scientific advisor of the EARL PET/CT accreditation program. Salim Kanoun is the founder of the Pixilib imaging CRO. This work was carried out under the auspices of the International Workshops on PET in Lymphoma and Myeloma. Data were presented in part at the workshop on October 6, 2023, held in Menton, France. No other potential conflict of interest relevant to this work was reported.

## Notes

KEY POINTS **QUESTION:** Can we measure TMTV in ^18^F-FDG PET/CT studies of lymphoma patients in a reliable and reproducible manner? **PERTINENT FINDINGS:** TMTV was measured reliably and reproducibly in 60 lymphoma cases from clinical trials by 12 international experts using a standardized segmentation method and workflow with freely available academic software. TMTV was easily measured in most cases with minimal reader interaction. The images and segmentations are provided as a benchmark dataset for PET readers, software developers, and vendors to estimate their ability to measure TMTV consistently with expected values. The benchmark can be used for comparison of the technical and clinical performance of other new segmentation methods. **IMPLICATIONS FOR PATIENT CARE:** TMTV is an established prognostic factor in lymphoma. Our proposed benchmark provides a standardized and practical measurement method for TMTV to facilitate its use as a reliable biomarker in patient care and clinical research.
